# Supplementary material for: Content-rich biological network constructed by mining PubMed abstracts
Source: BMC Bioinformatics. 2004 Oct 8;5:147. doi: 10.1186/1471-2105-5-147 (PMC528731; doi:10.1186/1471-2105-5-147)
Supplement: Additional File 5 — The original Chilibot query results of the term "long-term potentiation (LTP)" and 22 other terms, limiting the latest references analyzed to the years 1990, 1995, 2000, and 2004. [file 1471-2105-5-147-S5.bz2 › chilibotAdditionalFile5/ltp2004/html/PKA_TAU.html]

 


 **PKA** and **TAU** 
  
Found 63 abstracts in PubMed,  **30 abstracts were retrieved and analyzed**.  


---

 Search Google  |
 PDF files only 
|  EDU domain only 

---

**Interactive relationship** (e.g. stimulation, inhibition, etc)

- Treatment with a microtubule polymerizing agent taxol, an adenylyl cyclase inhibitor, or a protein kinase A  **PKA**  inhibitor, counteracted the effects of mutant  **tau**  on ICa.  Ref: 14511120 J Neurochem, 2003
- Calyculin A 0.1 microM inhibited approximately 50% PP 1, approximately 20% PP 2A, 50% GSK 3 and approximately 30% cdk5 but neither inhibited the activity of cyclin AMP dependent protein kinase A  **PKA**  nor resulted in the hyperphosphorylation of  **tau**  at any of the above sites.  Ref: 11086171 FEBS Lett, 2000
- Treatment of brain slices with 1 microM okadaic acid plus 0.1 microM calyculin A inhibited approximately 100% of both PP 2A and PP 1, approximately 80% of GSK 3, approximately 50% of cdk5 and approximately 30% of cdc2 but neither inhibited  **PKA**  nor resulted in the hyperphosphorylation of  **tau**  at any of the above sites.  Ref: 11086171 FEBS Lett, 2000
- Prior phosphorylation by PKC enhanced the nature and extent of AD like  **tau**  antigenicity generated by subsequent incubation with MAP kinase yet inhibited that generated by subsequent incubation with  **PKA** .  Ref: 10081606 Cell Mol Neurobiol, 1999
- Our results indicate that a  **tau**  mutation which decreases its microtubule binding ability augments calcium influx by depolymerizing microtubules and activating adenylyl cyclase and  **PKA** .  Ref: 14511120 J Neurochem, 2003
- At 3 6 h, kinase assays specific for  **PKA**  and CaMKII showed no increased activity toward either  **tau**  or selective substrates.  Ref: 11121021 Proc Natl Acad Sci U S A, 2000
- The treatment of  **tau**  by  **PKA**  and GSK 3 combination induced phosphorylation of  **tau**  at Ser 195, Ser 198, Ser 199, Ser 202, Thr 205, Thr 231, Ser 235, Ser 262, Ser 356, Ser 404.  Ref: 12894878 Chin Med Sci J, 2002
- 2 The treatment of  **tau**  by  **PKA**  and GSK 3 combination induced phosphorylation of  **tau**  at Ser 195, Ser 198, Ser 199, Ser 202, Thr 205, Thr 231, Ser 235, Ser 262, Ser 356, Ser 404.  Ref: 12903511 Zhongguo Yi Xue Ke Xue Yuan Xue Bao, 2000
- However, LLP  **TAU**  maintenance was completely suppressed by KT5720, an inhibitor of the cAMP dependent protein kinase  **PKA** .  Ref: 12559119 Neuropharmacology, 2003
- By contrast,  **PKA**  stimulated channels displayed a < ; 5% first pulse block and much slower block development  **tau** =405 85 seconds.  Ref: 12835205 Circulation, 2003
- From these data, we conclude that  **PKA**  phosphorylated  **tau**  retains the ability to bind tubulin and promote tubulin assembly.  Ref: 10449722 Proc Natl Acad Sci U S A, 1999
- **PKA**  phosphorylated  **tau**  binds to tubulin in the presence of TMAO and lowers the critical concentration of tubulin needed for assembly.  Ref: 10449722 Proc Natl Acad Sci U S A, 1999
- Phosphorylation of  **tau**  by casein kinase 1 CK 1, cyclic AMP dependent protein kinase  **PKA**  and glycogen synthetase kinase 3 GSK 3 separately inhibited its biological activity and the inhibition of this activity by CSK 3 was significantly increased if  **tau**  was prephosphorylated by CK 1 or  **PKA** .  Ref: 12894878 Chin Med Sci J, 2002
- Microinjection of an olfactory marker protein gene promoter driven double cassette vector directed the expression of both the dominant form of  **PKA**  and green fluorescent protein fused with the microtubule associated protein  **tau**  in the same olfactory neurons.  Ref: 12077193 J Neurosci, 2002
- 1 Phosphorylation of  **tau**  by casein kinase 1 CK 1, cyclic AMP dependent protein kinase  **PKA**  and glycogen synthetase kinase 3 GSK 3 differentially inhibited its biological activity, and the inhibition of this activity of  **tau**  by GSK 3 was significantly increased if  **tau**  was prephosphorylated by CK 1 or  **PKA** .  Ref: 12903511 Zhongguo Yi Xue Ke Xue Yuan Xue Bao, 2000
- Ser 262 in human recombinant  **tau**  protein is a markedly more favorable site for phosphorylation by CaMKII than  **PKA**  or PhK.  Ref: 9801171 FEBS Lett, 1998
- Phosphorylation of  **tau**  by  **PKA**  plus GSK 3 at Thr 205 might play a key role in  **tau**  pathology in AD.  Ref: 12894878 Chin Med Sci J, 2002
- Here, the human brain  **tau**  with 441 amino acids was phosphorylated by cyclic AMP dependent protein kinase  **PKA**  or glycogen synthase kinase 3beta.  Ref: 10449722 Proc Natl Acad Sci U S A, 1999
- The cAMP dependent protein kinase  **PKA**  has been implicated in the Alzheimer s disease pathology of abnormal  **tau**  phosphorylation leading to neurofibrillary tangle NFT formation, as well as in amyloid precursor protein alpha secretase processing.  Ref: 10082824 Brain Res, 1999
- Between 5 HT1A agonists wide differences in in vivo affinity and efficacy were observed, with values of the  **pKA**  ranging from 5.67 for flesinoxan to 8.63 for WAY 10 35 and of the Log  **tau**  ranging from 1.27 for WAY 10 35 to 0.62 for R 8 OH DPAT.  Ref: 14718609 J Pharmacol Exp TherJ Pharmacol Exp Ther, 1999

**Parallel relationship** (e.g. studied together, co-existance, homology, etc.)

- By use of the method of partial receptor alkylation and analysis of concentration effect curve data by direct, operational model fitting methods, the affinity  **pKA**  and efficacy  **tau**  were 5.74 0.07 and 7.50 1.05, respectively n = 6.  Ref: 9283709 Br J Pharmacol, 1997
- We have studied the relationship between the phosphorylation of  **tau**  by several kinases MARK,  **PKA** , MAPK, GSK3 and its assembly into PHFs.  Ref: 10090741 Biochemistry, 1999
- **PKA**  phosphorylated  **tau**  2.7 mol phosphates mol does not promote tubulin assembly as judged by spectrophotometric and atomic force microscopy measurements, unless trimethylamine N oxide TMAO, a natural occurring osmolyte, is included in these assays.  Ref: 10449722 Proc Natl Acad Sci U S A, 1999
- In all cases, the model converged and estimates of apparent affinity  **pKA**  and efficacy  **tau**  were obtained which were highly consistent with results from in vitro radioligand binding studies.  Ref: 9352393 Int J Clin Pharmacol Ther, 1997
- The most potent inhibition was seen by a combined phosphorylation of  **tau**  with  **PKA**  and GSK 3.  Ref: 12903511 Zhongguo Yi Xue Ke Xue Yuan Xue Bao, 2000
- The most potent inhibition was seen by a combined phosphorylation of  **tau**  with  **PKA**  and GSK 3.  Ref: 12894878 Chin Med Sci J, 2002
- The resulting concentration effect relationships were subsequently analyzed on the basis of the operational model of agonism to estimate the operational affinity  **pKA**  and efficacy Log  **tau**  at the 5 HT1A receptor in vivo.  Ref: 14718609 J Pharmacol Exp TherJ Pharmacol Exp Ther, 2002
- In this study we compared the phosphorylation of  **tau**  at Ser 262 by CaMKII, PhK and  **PKA**  in vitro as determined by radioimmunoblots developed by the monoclonal antibody 12E8 which recognizes P Ser 262 and P Ser 356.  Ref: 9801171 FEBS Lett, 1998
- The individual and sequential influence of protein kinase C PKC, protein kinase A  **PKA**  and mitogen activated protein kinase MAP kinase on human brain  **tau**  was examined.  Ref: 10081606 Cell Mol Neurobiol, 1999
- We identified among these proteins an 8 0 kDa and the  **tau**  protein as specific substrates for protein kinase A  **PKA** , MARCKS, and neurogranin as specific substrates for protein kinase C PKC, and prostaglandin D synthase as substrate for casein kinase II CKII.  Ref: 10226777 Hippocampus, 1999
- Modelling Alzheimer specific abnormal  **Tau**  phosphorylation independently of GSK3beta and  **PKA**  kinase activities.  Ref: 11959122 FEBS Lett, 2002
- cAMP dependent protein kinase phosphorylations on  **tau**  in Alzheimer s disease.To elucidate the role cAMP dependent protein kinase phosphorylations on  **tau**  play in Alzheimer s disease, we have generated highly specific monoclonal antibodies, CP 3 and PG 5, which recognize the  **PKA**  dependent phosphorylations of ser214 and ser409 in  **tau**  respectively.  Ref: 10460255 J Neurosci, 1999
- Further analysis demonstrates that the beta catalytic subunit of  **PKA** , the beta II regulatory subunit of  **PKA** , and the 79 kDa A kinase anchoring protein, are tightly associated with the neurofibrillary pathology, positioning cAMP dependent protein kinase to participate directly in the pathological hyperphosphorylation of  **tau**  seen in Alzheimer s disease.  Ref: 10460255 J Neurosci, 1999
- **PKA**  phosphorylations on  **tau**  developmental studies in the mouse. **PKA**  phosphorylations of  **tau**  may be an early event in the development of neurofibrillary pathology in Alzheimer s disease.  Ref: 10965152 Dev Neurosci, 2000
